# Risk, lifestyle and non-communicable diseases of poverty

**DOI:** 10.1186/s12992-023-00914-z

**Published:** 2023-03-02

**Authors:** Lenore Manderson, Sara Jewett

**Affiliations:** 1 School of Public Health, University of the Witwatersrand, Johannesburg, South Africa; 2 School of Social Sciences, Monash University, Clayton, Australia; 3 Health & Society Division, School of Public Health, University of the Witwatersrand, Johannesburg, South Africa

**Keywords:** Non-communicable diseases, Non-communicable diseases of poverty, LMIC, Commercial determinants, Structural determinants, Health inequities, Nutritional transition, Obesity, Diseases of lifestyle

## Abstract

Common discourse in public health and preventive medicine frames non-communicable diseases, including cardiovascular and metabolic diseases, as diseases of ‘lifestyle’; the choice of terminology implies that their prevention, control and management are amenable to individual action. In drawing attention to global increases in the incidence and prevalence of non-communicable disease, however, we increasingly observe that these are non-communicable diseases of poverty. In this article, we call for the reframing of discourse to emphasize the underlying social and commercial determinants of health, including poverty and the manipulation of food markets. We demonstrate this by analysing trends in disease, which indicate that diabetes- and cardiovascular-related DALYS and deaths are increasing particularly in countries categorized as low-middle to middle levels of development. In contrast, countries with very low levels of development contribute least to diabetes and document low levels of CVDs. Although this might suggest that NCDs track increased national wealth, the metrics obscure the ways in which the populations most affected by these diseases are among the poorest in many countries, and hence, disease incidence is a marker of poverty not wealth. We also illustrate variations in five countries — Mexico, Brazil, South Africa, India and Nigeria — by gender, and argue that these differences are associated with gender norms that vary by context rather than sex-specific biological pathways.

We tie these trends to shifts in food consumption from whole foods to ultra-processed foods, under colonialism and with continued globalization. Industrialization and the manipulation of global food markets influence food choice in the context of limited household income, time, and household and community resources. Other factors that constitute risk factors for NCDs are likewise constrained by low household income and the poverty of the environment for people with low income, including the capacity of individuals in sedentary occupations to engage in physical activity. These contextual factors highlight extremely limited personal power over diet and exercise. In acknowledging the importance of poverty in shaping diet and activity, we argue the merit in using the term non-communicable diseases of poverty and the acronym NCDP. In doing so, we call for greater attention and interventions to address structural determinants of NCDs.

## Background

The framing of non-communicable diseases (NCDs) as ‘lifestyle’ diseases shapes discourses of personal responsibility and blame. Lifestyle ‘choices’ are loosely represented in terms of choice in relation to residence, diet, leisure and so on, and are emphasized as the dominant contributing factors for cardiometabolic disease, including obesity, cardiovascular diseases, and metabolic diseases such as type 2 diabetes. Other behaviors, including alcohol consumption and smoking, are also implicated in NCDs, including various cancers. Attention to lifestyle factors suggests that these conditions and diseases can be averted, and their complications and co-morbidities prevented, by individual behavioral change including weight loss, exercise, and a ‘healthy’ diet, however defined.

The emphasis on lifestyle assumes personal volition and the capacity of individuals to avoid risk factors or to modify those already identified, and to make healthy decisions regarding food choice and intake, level of exercise and ideal weight [1]. Failure to avert risk through behavioral change implies lack of self-control and willpower. In this neoliberal narrative, differences between individuals and societies, in different social, cultural and economic settings, are ignored or minimized. This is an attractive approach; like any neoliberal policy, it places responsibility on individuals and obviates state responsibility, including fiscal policy and the allocation of services and resources. The individualization of risk factors also ignores the industries and other structural factors that directly contribute to the growing global burden of NCDs, and occludes the political, economic, commercial and social determinants of health that underpin the proliferation of NCDs and other health problems globally [2, 3].

We join a number of colleagues in calling for the reframing of discourse to emphasize the underlying causes of non-communicable diseases [4, 5]. In the following, we continue this conversation of the need to consider the commercial and social determinants of health, particularly poverty, and to move these to the fore by explicitly acknowledging that these are non-communicable diseases of poverty (we use the acronym NCDP below). The inclusion of *poverty* in this umbrella term signifies how low income and limited resources at individual, household and community levels, and associated personal powerlessness and lack of choice, combine to predetermine and constrain individual options of diet, nutrition and activity.

In reviewing the distribution of NCDPs globally and within specific countries, using gender-disaggregated data from the Global Burden of Disease study, we show how these diseases trace social and economic fault lines. We draw attention to the limitations of individually focused interventions which fail to address the structural and commercial determinants of health. We dismantle assumptions of ‘choice’ embedded in most NCD prevention strategies. In this context we build on major review documents and critical responses to them, including the EAT-Lancet Commission on Food in the Anthropocene [6, 7] and the Lancet Commission report on The Global Syndemic of Obesity, Undernutrition, and Climate Change [8, 9].

## Dispensing with ‘lifestyle’ as cause

In 2022, around 13.6% of the world’s population were living in extreme poverty, surviving on United States (US) $2.15 (PPP) per day, with around two thirds of this population living in countries on the continent of Africa. The estimated poverty line in US dollars for lower middle-income countries (LMICs) is now $3.65 and for upper middle-income countries (UMICs) $6.85 [10]. Rising numbers of countries have moved from low to lower-middle, middle and upper-middle income, reflecting gross domestic product (GDP), but this ignores concurrent widening inequality. For example, South Africa is categorized as an upper-middle income country, but the country also has the greatest inequality in the world [11], and one third of this population lives on or below the poverty line. In this one country, therefore, 22 million people have little choice of residence, education, employment, health care, or food. The Covid-19 pandemic and more recent global economic stressors have led to increases in the cost of basic food items and other household products, resulting in growing increases in all poverty indices [12–14].

Diseases of lifestyle suggest both agency and privilege. Agency asserts the capacity of individuals to exercise choice, but this is primarily possible only for those who already enjoy social, economic and political power. Agency therefore flows from power. People with relatively high incomes have greater ability than those who are cash poor to modify ‘lifestyle’: to negotiate access to markets and services; to choose the areas in which they reside and the quality of their housing and its environs; to access and make sense of health information; to make dietary decisions that are not constrained by the market conditions of food choices; and to have the time and opportunities to exercise. People who are desperately poor are especially powerless, and accordingly they lack agency to make food and dietary choices. This is often exacerbated by the non-availability and unaffordability of quality foods. Moreover, large numbers of the world’s population live in overcrowded and unsanitary environments where food storage may be difficult and where they may share cooking facilities. The poorest employed people worldwide undertake physical demanding work, as unskilled laborers and contract workers in agriculture, manufacturing and extraction industries; this population is not sedentary, and the injunction to exercise makes little sense. On the other hand, poor people in sedentary occupations typically lack the time and resources to exercise, and lack local security to exercise outdoors. Poverty is not a lifestyle choice.

Worldwide, extensive poverty within and across communities and states, and between nations, shapes food production, distribution and consumption, influencing and largely predicting who are most likely to suffer from NCDs, the development of comorbidities and complications, and the risk of early death. These same populations vulnerable to NCDs also bear the heaviest burden of infectious disease, injuries, and continued poor maternal and child health, hence the emphasis on the ‘quadruple burden’ of disease [15–17]. Individual and national poverty, in relation to cash and to public services, contribute to disparities within and between countries in terms of life expectancy, quality of life and morbidity. Hence these are, predominantly, NCDPs.

## The inequitable distribution of NCDPs

Globally, there is an increasing incidence of NCDPs, including cardiovascular diseases (mainly heart disease and stroke), diabetes, osteoarthritis, and various cancers (breast, prostate, liver, kidney, and colon) [18]. Once characterized as indexing an epidemiological transition from infectious to non-communicable diseases, as noted above these diseases co-occur with continued high levels of various infectious diseases, the latter often associated with poorly maintained or limited infrastructure, inadequate housing, crowding and unsanitary conditions. While this is particularly so in poor countries, it is also the case among poor communities in high-income settings.

The distribution of NCDP mortality is grossly inequitable, with 86% of NCD-attributable premature deaths before age 70 located in LMICS [16–18]. Despite global declines in incidence, cardiovascular diseases (CVDs) are the main contributors to NCD mortality, and diabetes mortality has increased globally over the past three decades in all regions. This can be seen in annual changes in attributable disability adjusted life years (DALYs) as well as mortality rates per 100,000 between regions with different development profiles (Table 1).

**Table 1:** Annual changes in diabetes and cardiovascular disease DALYs and mortality rates, 1990–2018, by level of development

Socio-demographic Index (SDI)	Annual Rate of Change between 1990 and 2018 mortality (per 100,000)					
	Diabetes – All	Diabetes – Male	Diabetes - Female	CVD - All	CVD – Male	CVD - Female
*Overall DALYs*	*.71 (.63, .79)*	*.82 (.72, .91)*	*.61 (.51.71)*	*−.03 (−.09, .03)*	*.02 (−.06, .10)*	*−.08 (−.15, −.01)*
Deaths	.62(.52, .72)	.76(.63, .89)	.51 (.38, .63)	.06 (.00, .13)	.11 (.03, .20)	.02 (−.07, .10)
*Low SDI - DALYs*	*0.15 (.04, .25)*	*0.10 (−.30, −.08)*	*.21 (.09, .35)*	*−.20 (−.27, −.11)*	*−.20 (−.30, −.08)*	*−.19 (−.28, −.06)*
Deaths	.05 (−.07, .17)	−.02 (−.16, .15)	.14 (−.02, .34)	−.14 (−.22, −.05)	−.17 (−.27, −.04)	−.10 (−.23, .06)
*Low-Middle – DALYS*	*.88 (.74, 1.02)*	*.92 (.76, 1.04)*	*.84 (.65, 1.04)*	*.13 (.03, .24)*	*.15 (.03, .29)*	*.11 (−.03, .25)*
Deaths	.90 (.70, 1.11)	.89 (.64, 1.21)	.90 (.63, 1.21)	.29 (.16, .41)	.28 (.14, .43)	.30 (.11, .49)
*Middle - DALYS*	*1.04 (.94, 1.14)*	*1.19 (1.06, 1.32)*	*.90 (.76, 1.04)*	*.20 (.10, .32)*	*.30 (.15, .47)*	*.09 (−.04, .22)*
Deaths	.71 (.63, .79)	.82 (.72, .91)	.61 (.51, .71)	.41 (.28, .55)	.51 (.33, .71)	.31 (.14. .49)
*High-Mid DALYS*	*.65 (.56, .73)*	*.81 (.71, .91)*	*.52 (.41, .60)*	*−.04 (.02, −.11)*	*0.00 (−.09, .10)*	*−.10 (−.02, −.17)*
Deaths	.51 (.40, .62)	.71 (.55, .86)	.38 (.25, .52)	.08(.00, .15)	.13 (.03, .23)	.04(−.04, .13)
*High - DALYS*	*.59 (.49, .68)*	*.76 (.67, .85)*	*.42 (.31, .50)*	*−.28 (−.31, −.26)*	*−.27 (−.30, −.25)*	*−.29 (−.33, −.26)*
Deaths	.19 (.14, .24)	.44 (.38, .49)	.02 (−.06, 07)	−.20 (−.25, −.17)	−.19 (−.23, −.17)	−.20 (−.27, −.16)

Data Source: *Institute for Health Metrics and Evaluation. Used with permission. All rights reserved*. https://vizhub.healthdata.org/gbd-results/ Accessed 8 October 2022

A closer analysis of trends over time indicates that countries falling in the low-middle to middle levels of development contribute most to increases in diabetes-related DALYs and mortality; in these same development regions, CVD-related DALYS and deaths are still increasing. This suggests that national income status, as reflected by GDP, for instance, are not the key issue in explaining NCD morbidity and mortality distributions, although this metric remains useful as indicating national capacity to manage health commitments and threats. One way of interpreting the continued and increasing importance of cardiometabolic diseases in LMICs is related to significant resource constraints on health services and medical care. But economic constraints have effects also at individual and household levels, including in relation to the living conditions and health care options of poorer populations. For example, affordable food items are limited for people on low incomes, and this likely affects more people in LMIC than in very low income settings, where subsistence farming and gardening may still contribute to household food supply. Countries with very low levels of development contribute least to diabetes and document low levels of CVDs, likely because of lower levels of urbanization and dependence on local food resources. In contrast, food choice limitations at local outlets and transport costs (to purchase cheaper and better quality food) affect poor households, contributing to poor health. In this context, life expectancy in China has increased where, in addition to improved access to and affordability of health care, extreme poverty has been redressed [19]. Further analysis of this pattern will be helpful to understand these variations.

How does this hypothesis work at a country level? Within regions that have the same level of development, as indicated by WHO’s designation by per capita income, we see varied patterns of attributable DALYs and mortality. In Table 2, we illustrate these variations for five countries — Mexico, Brazil, South Africa, India and Nigeria — for this same time period and, because of established disparities, by gender. In all cases excepting Nigeria, these countries contribute more than the global average to NCD morbidity and mortality. But why should there be such differences between countries? One explanation is their position in relation to nutritional transitions from whole foods to the consumption of more ultra-processed foods (UPFs). An extreme example of this, as related by Vorster and colleagues [20], was that in 2008 the average world consumption of Coca-Cola was 85 servings per person per year, while in Nigeria it was only 27 servings and in South Africa a whopping 252 [20]. These metrics indicate that countries are at very different stages in their nutritional transitions and the penetration of global food markets.

**Table 2:** Annual changes in diabetes and cardiovascular disease DALYs and mortality rates, 1990–2018, in five middle-income countries

Country	Annual Rate of Change between 1990 and 2018 mortality (per 100,000)						WHO 2022 designation & Gini Index
	Diabetes – All	Male	Female	CVD - All	Male	Female	
Mexico – Deaths	.89 (.65, 1.14)	1.20 (.82, 1.66)	.64 (.38, .96)	.58 (.40, .78)	.61 (.34, .94)	.56 (.23, .82)	Upper-middle income (Gini: 45.4)
DALYS	.85 (.69, 1.02)	1.10 (.85, 1.39)	.65 (.47, .84)	.38 (.21, .56)	.49 (.23, .80)	.26 (.08, .46)	
South Africa - Deaths	1.15 (.93, 1.38)	1.20 (.91, 1.51)	1.13 (.86, 1.41)	.28 (.18, .40)	.23 (.10, .37)	.33 (.21, .48)	Upper-middle income (Gini: 63.0)
DALYs	.99 (.85, 1.14)	1.07 (.87, 1.30)	.94 (.76, 1.12)	.07 (.00, .15)	.09 (−.02, .21)	.05 (−.04, .16)	
Brazil - Deaths	.65 (.55, .73)	.83 (.71, .96)	.51 (.40, .61)	.01 (−.04, .06)	−.01 (−.08, .04)	.05 (−.03, .10)	Upper-middle income (Gini: 53.4)
DALYS	.58 (.51, .65)	.70 (.62, .79)	.48 (.39, .57)	−.13 (−.28, .11)	−.14 (−.37, .18)	−.05 (−.27, .23)	
India - Deaths	1.13 (.78, 1.59)	1.06 (.58 (1.70)	1.22 (.70, 1.22)	.32 (.11, .52)	.25 (.01, .54)	.42 (.10, .78)	Lower-middle income (Gini: 35.7)
DALYS	1.08 (.87, 1.30)	1.06 (.78, 1.37)	1.11 (.82, 1.46)	.14 (−.04, .31)	.11 (−.10, .38)	.18 (−.07, .46)	
Nigeria - Deaths	−.16 (−.36, .10)	−.13 (−.42, .29)	−.18 (−.43, .13)	−.42 (−.59, −.22)	−.39 (−.08, .04)	−.45 (−.65, −.21)	Lower-middle income (Gini: 35.1)
DALYS	−.10 (−.28, .11)	−.14 (−.37, .18)	−.05 (−.27, .23)	−.40 (−.56, −.21)	−.40 (−.59, −.14)	−.39 (−.58, −.13)	

Data Source: *Institute for Health Metrics and Evaluation. Used with permission. All rights reserved*. https://vizhub.healthdata.org/gbd-results/ Accessed 17 October 2022

## Structures of exclusion

Below, we focus on the nutritional transitions occurring within poor countries and communities, but first, we reflect on the clear gender differences in NCDPs. These differences are evident in Tables 1 and 2, especially in terms of rates of change in diabetes morbidity and mortality. These patterns are not consistent by country or development level. Examination of patterns of diseases by sex and gender has been underrepresented in NCD research [21], both with respect to CVDs [22] and diabetes [23]. While some differences may relate to sex-specific biological pathways, there are compelling arguments that many differences are associated with gender, including differences in financial pressures, psychological stresses, food choice and allocation within households, and possibly gender norms related to eating behavior and food choice [21]. A gender analysis of NCDP risk needs to be conducted with attention to country-specific contexts and to country or community poverty profiles. The recent work of Magodoro and colleagues [24] in Uganda illustrates this point, where their sex-disaggregated findings contradict global trends; but also, although rural women had better measures of ideal cardiovascular health (CVH) behaviors than their male counterparts, they still had a worse overall ideal CVH profile. Gender-specific research has been conducted with US and United Kingdom (UK) populations (and so high-income countries) [25–27], but further research is needed in this area.

We included the Gini Index for select middle income countries in Table 2 to draw attention to how average income used alone can mask the extent of poverty for large segments of a population, and so prevent enquiry into how poverty operates. The table includes the country with the highest inequality in the world (South Africa) in contrast to two countries with relatively low income inequality (India and Nigeria). The inequitable distributions of NCDs between countries with similar development profiles (see Table 1) are replicated within countries (Table 2). For instance, historically NCDs have been framed as diseases of affluence and voraciousness. However, even in the United States, a high-income country, persistent income- and education-related disparities are well documented for diabetes [25] and CVDs [26], with poor people disproportionately affected. Yet as Beaglehole, Reddy and Leeder argued 15 years ago [27], there is still a need to include CVD in what they characterize as the ‘global development agenda’. Cardiometabolic diseases are both causes and consequences of poverty, imposing substantial health and economic costs at household and state levels in LMICs. Yet in the discourse of disease, many researchers and policy makers continue to treat lifestyle and choice as most significant. Accordingly, lifestyle choices rather than change at the systems level have been the focus of health promotion.

Within affluent countries, NCDs are disproportionately experienced in poorer communities, within which households and communities experience systemic marginalization that impacts on income generation, security of employment, food security and access to health care. The patterns of NCD distribution track the intersections between gender, race and social status. In a cross-sectional survey in the US, for example, Blacks in the mid-South region were disproportionately affected by chronic health conditions and these were also linked to income disparities [28]. These patterns relate not only to the distribution of NCDs, but also to the management of disease and access to quality care. This is also the case for type 2 diabetes, where living in the South, being indigenous, Black or Asian, uninsured and low income were all associated with poorer management [29]. In many LMICs, sexism and racism intersect with the vestiges of colonialism and ongoing globalization to shape daily living, health inequalities and health outcomes [30]. Dramatically different population-level NCD morbidity and mortality trajectories follow the fractures of poverty, structural violence and development profiles, whether seen through the lens of regional or country development or within country inequities. This is true also for NCDs linked to infections and environmental exposures [31, 32].

## Blaming obesity

If modifiable behaviors are the proximate cause of NCDs in individuals, as dominates the discourse, we need to ask why these behaviors are so strongly correlated with poverty and interrogate the relationship between the two. Is it more likely that people do not consume nutritious diets because of constrained income choices or that their choice of unhealthy diets and ongoing health problems have led them into poverty? While we recognize the compounding and interactive effects of health and poverty [33], the former is strongly supported by global evidence.

More people die from health conditions associated with being overweight or obesity than they do from being underweight. The WHO fact sheet on overweight and obesity [34] documents that worldwide, in 2016, 39% of adults aged 18 years and over were overweight, and 13% were obese. The prevalence of obesity, as measured by BMI, nearly tripled between 1975 and 2016, and there were more people overweight than underweight worldwide except in parts of sub-Saharan Africa and Asia (Ethiopia, Niger, Senegal, India, Bangladesh, Myanmar, and Cambodia) [35]. These data reflect the emphasis on weight in global strategic documents and reports this century. At the same time, these documents link overweight and obesity explicitly to individual behaviors, and advocate health promotion approaches to target these behaviors.

For example, the WHO Global Strategy on Diet, Physical Activity and Health [36], adopted in 2004 and re-endorsed in 2011, recommended public health action to support healthy eating and regular physical activity. While the strategy drew attention to the role of transnational food and beverage corporations in marketing foods rich in sugar, salt and fats, particularly to children, it also called on member states to encourage ‘healthy food choices’. The strategy itself paid little attention overall to the economic, political and commercial determinants of health: obesity and overweight were primarily represented as the result of poor individual eating patterns, ill-informed decisions about diet and nutrition, and exercise (or its absence). The EAT-Lancet Commission on Food in the Anthropocene [7] and the Lancet Commission on The Global Syndemic of Obesity, Undernutrition, and Climate Change [9] replicate this focus on the individual. Both commissions acknowledge the health effects of climate change, and yet focus in on individual behavior when proposing action.

The term syndemics captures the interactions of biology and social factors, including the synergies of health and illness and the pathologies of particular health conditions. It provides a gloss of the relationship, in Merrill Singer’s classic example, between poverty, sex work, HIV and TB [37]. In the Lancet Commission on the Global Syndemic [9], as the title suggests, syndemic refers to the intersections of diet, food availability, climate change, body weight, vulnerability, and the development of disease. The Commission, lead by Boyd Swinburn, highlights the role of obesogenic environments, that is, economic, environmental and other factors which restrict the availability of quality foods and, by shaping food choice, so promote obesity. Individuals find themselves living in ‘food deserts’ where the promotion of ‘healthy food choices’ makes a mockery of access to such products. Yet despite this, Swinburn and colleagues highlight the “personal agency individuals have in making their choices from the environments available … the influence the environment has on those choices … (and) the influence that the individual has on changing the environments and systems around them” (804). Swinburn and colleagues acknowledge that conditions of poverty restrict people’s capacity to make healthy choices, but even so, the constraints in obesogenic settings are not considered immutable:

People can act as agents of change in their roles as elected officials, employers, parents, customers, and citizens and influence the societal norms and institutional policies of worksites, schools, food retailers, and communities to address The Global Syndemic … the collective influence of individuals, civil society organisations, and the public can stimulate the reorientation of human systems to promote health, equity, economic prosperity, and sustainability (792).

In response, we question the existence of choice in environments dominated by fast food outlets, where available fresh food is limited and expensive, where people buy small quantities of inexpensive food on a daily basis, and where lack of neighborhood safety and other environmental factors (poor transport, urban design and land use) limit physical activity. In doing so, we align ourselves with the authors of a recent systematic review which explored a direct relationship between food insecurity and chronic diseases; they called for more research, particularly in LMIC, into ‘the systematic effects of poverty’ leading to chronic diseases [38], after finding that the popular explanation of obesity causing NCDs did not withstand scrutiny. We also align ourselves to the commentary from the Nutrire CoLab [6] collective on the EAT Commission. For although the commission acknowledged that human activities have caused climate change, deforestation and biodiversity loss, with deleterious effects on food supply, variety, affordability and consumption, it still emphasized ‘lifestyle’ as a core factor for diet-related diseases and slipped over structural inequality to explain limits to diet.

## Food transitions

To reinforce our argument for NCDPs, we step back at this point to reflect on the nutritional transition, from whole foods to ultraprocessed foods (UPFs), across the globe. This occurred throughout the twentieth century, in association with colonialism and globalization. However, in many countries, the nutritional transition has been relatively recent and rapid. This has been the result of a toxic mix of changes in available and affordable quality foods, the unfettered marketing of commercial UPFs and sugar sweetened beverages (SSBs), and the promotion of certain foods as modern, sophisticated and aspirational, and in contrast to this, the effects of limited household income, time and other resources to provide nutritious meals. This is true not only in middle income countries, but also in high income settings where people surviving on social welfare or casual labor, and living in shared accommodation, cars and mobile homes, often lack food storage and cooking facilities, and lack the cash to purchase food other than on a day-to-day basis. Fried take-away food is often the cheapest and most filling option available to families which are cash, time, and resource poor [1, 39].

The displacement of traditional foods with UPFs and SSBs was in part the consequence first of the development of colonial plantations and slavery, as illustrated by the colonial production of food commodities like coffee and sugar [40, 41] and non-food items such as cotton and rubber, then with the introduction and expansion of monocropping, commercial fishing and exploitative working conditions [42–44]. Repressive imperial regimes forced dietary change, often — as in the case of settler colonies — paying people for their labor with white sugar, white flour and tea, rice and tinned meat, or, in South Africa, with wine [45]. Forced dietary change escalated with globalization in the second half of the twentieth century, and in the past seven decades through global trade pacts in agricultural goods, foreign direct investments in food processing, and commercial marketing shifts to niche (poorer) markets, as meticulously documented by Corrina Hawkes in 2006 [46]. As she showed, the rise of obesogenic environments resulted in rapid increases in chronic disease. There was a marked shift from diabetes and heart disease being diseases of affluence and gluttony (‘lifestyle’) to diseases of poverty of cash, time, security and place.

In their landmark research, Zimmet and colleagues identified high rates of coronary heart disease and diabetes with shifts from traditional diets to poor quality replacement foods, including refined sugar, refined flour, white rice, tinned fish, and cheap tinned and processed meat (e.g. Spam) [47]; shifts in diet often increased with the colonial and commercial appropriation of food resources [48, 49]. Large scale exploitation of food resources, for example of fishing in the Pacific, and trade agreements favoring the global north [50], have further forced food insecurity on poor populations and nations. Various claims have been made about different peoples as ‘the most obese’ in the world — the Pima, Pacific island populations, Mexicans — with little attention to the household and national economics that have driven this transition.

## Conclusion

The WHO STEPwise approach to NCD risk factor surveillance [50] highlights the importance of collecting data on biological risk factors, including overweight and obesity, and on tobacco use, alcohol use, physical inactivity and unhealthy diet. In this as in other guidelines to address the causes of and reduce the incidence of NCDs, little attention is given to tracking the social and commercial determinants of health, even less to environmental determinants such as air pollution and occupational exposures.

We are not arguing against the body of evidence that has linked individual behaviors to NCDs. We acknowledge that personal factors, such as sedentary work and intake of foods high in refined carbohydrates, sugar and oil, contribute to the clinical development of cardiometabolic disease and increases in incidence and prevalence. We also note that the growing diagnosis of NCDs able to be managed by ‘drugs for life’ has its own trajectory - biomedicalization and pharmaceutical management are also globalized [51–53]. We call for greater emphasis and a closer interrogation of the structural and commercial factors that underlie such behaviors, both through adopting the NCDP frame and acronym and through closer measurement of these dynamics. Our argument is that NCDPs are not the outcomes of lifestyle ‘choices’ so much as a consequence of the systems that produce limited behavioral options. The individualization of disease through emphasis on lifestyle as an etiology minimizes the predominant role of poverty at multiple levels, from individual to the state, to explain the patterning of disease. The challenge, for academic and policy researchers and governments, is to find ways to effectively reduce poverty while addressing the powerful social and commercial determinants that impact individual diets and health outcomes.

## Data Availability

The datasets used to generate Tables 1 and 2 are from the Global Burden of Disease Collaborative Network, Global Burden of Disease Study 2019 (GBD 2019) Results (2020, Institute for Health Metrics and Evaluation – IHME): https://vizhub.healthdata.org/gbd-results/

## Abbreviations

BMI: Body Mass Index
CVD: Cardiovascular disease
CVH: Cardiovascular health
DALYs: Disability Adjusted Life Years
GDP: Gross Domestic Product
LMIC: Low and middle income countries
NCD: Non-communicable disease
NCDP: Non-communicable disease of poverty
SSB: Sugar sweetened beverages
UMIC: Upper middle income country
UPF: Ultraprocessed foods
US: United States
UK: United Kingdom
WHO: World Health Organization
